# High CO_2_/hypoxia-induced softening of persimmon fruit is modulated by DkERF8/16 and DkNAC9 complexes

**DOI:** 10.1093/jxb/eraa009

**Published:** 2020-01-12

**Authors:** Wei Wu, Miao-miao Wang, Hui Gong, Xiao-fen Liu, Da-long Guo, Ning-jing Sun, Jing-wen Huang, Qing-gang Zhu, Kun-song Chen, Xue-ren Yin

**Affiliations:** 1 Department of Horticulture, Zhejiang University, Hangzhou, Zhejiang, China; 2 Zhejiang Provincial Key Laboratory of Integrative Biology of Horticultural Plants, Hangzhou, Zhejiang, China; 3 College of Horticulture, Henan Agricultural University, Henan, China; 4 College of Forestry, Henan University of Science and Technology, Luoyang, Henan, China; 5 College of Resources and Environment Sciences, Baoshan University, Baoshan, Yunnan, China; 6 CONICET- National University of La Plata, Argentina

**Keywords:** *DkEGase1*, NAC, NAC-ERF complex, persimmon fruit, post-deastringency softening, transcriptomic analysis, transcriptional regulation

## Abstract

Most persimmon (*Diospyros kaki*) cultivars are astringent and require post-harvest deastringency treatments such as 95% CO_2_ (high-CO_2_ treatment) to make them acceptable to consumers. High-CO_2_ treatment can, however, also induce excessive softening, which can be reduced by adding 1-methylcyclopropene (1-MCP). Previous studies have shown that genes encoding the ETHYLENE RESPONSE FACTORS (ERFs) DkERF8/16/19 can trans-activate *xyloglucan endotransglycosylase/hydrolase* (*DkXTH9*), which encodes the cell wall-degrading enzyme associated with persimmon fruit softening. In this study, RNA-seq data between three treatments were compared, namely high-CO_2_, high-CO_2_+1-MCP, and controls. A total of 227 differentially expressed genes, including 17 transcription factors, were predicted to be related to persimmon post-deastringency softening. Dual-luciferase assays indicated that DkNAC9 activated the *DkEGase1* promoter 2.64-fold. Synergistic effects on transcription of *DkEGase1* that involved DkNAC9 and the previously reported DkERF8/16 were identified. Electrophoretic mobility shift assay indicated that DkNAC9 could physically bind to the *DkEGase1* promoter. Bimolecular fluorescence complementation and firefly luciferase complementation imaging assays indicated protein–protein interactions between DkNAC9 and DkERF8/16. Based on these findings, we conclude that DkNAC9 is a direct transcriptional activator of *DkEGase1* that can co-operate with DkERF8/16 to enhance fruit post-deastringency softening.

## Teaser

High CO_2_ triggers post-deastringency softening in persimmon fruit through interactions between ethylene response factors and NAC9 that lead to activation of the cellulose-degrading gene *DkEGase1*.

## Introduction

Texture, usually reflected by firmness, is critical for fruit storability and quality (Brummell, 2006; Li *et al.*, 2010; Wang *et al.*, 2018). Fruit texture is not only dependent on the genetic background, but is also influenced by external environments, such as temperature (Yang *et al.*, 2007) and plant hormones (Li *et al.*, 2017*b*; Zhang *et al.*, 2018). Low-oxygen environments, usually applied in controlled atmospheres, have been used by industry and researchers to efficiently prolonging post-harvest storage and maintenance of firmness in various fruit (McDonald and Harman, 1982; Siddiqui *et al.*, 1996; Yahia, 1998). A particular side effect of high-CO_2_ (95% CO_2_, 1% O_2_, 4% N_2_) treatment is the accelerated removal of astringency in persimmon (*Diospyros kaki*) fruit. Although this greatly improves the taste for consumers, it subsequently triggers rapid softening (Wang *et al.*, 2017), which is undesirable. Persimmon fruit therefore provides an interesting model to investigate changes in fruit flavor and texture in response to hypoxia. In many fruits, ripening and quality-related gradients of metabolites can occur in relation to *in situ* hypoxic areas generated inside the fruit (Pedreschi *et al.*, 2009; Biais *et al.*, 2010). In persimmon, there is a burst in acetaldehyde production under low oxygen, which contributes to deastringency by precipitating the soluble tannins responsible for the astringent taste (Taira *et al.*, 2001; Min *et al.*, 2012; Zhu *et al.*, 2018). It is known that production of acetaldehyde under anoxia is regulated by various transcription factors (TFs) for example, ethylene response factors (*ERF*s; Min *et al.*, 2012; Zhu *et al.*, 2018) and *WRKY* (Zhu *et al.*, 2019). However, the mechanism(s) regulating the excessive softening that occurs after deastringency treatment are poorly understood.

Persimmon is a climacteric type of fruit, showing an increase in ethylene production during softening (Harima *et al.*, 2003; Nakatsuka *et al.*, 2011; Yin *et al.*, 2012). As with other fruit produced for consumption, modulations in taste, softening, and texture are the main post-harvest issues. In persimmon, the activities of several enzymes have been shown to be positively correlated with fruit softening, namely polygalacturonase (PG: endo-type, EC 3.2.1.15; exo-type, EC 3.2.1.67), pectin methylesterase (PME, EC 3.1.1.11), β-galactosidase (β-gal, EC 3.2.1.23), and xyloglucan endotransglycosylase/hydrolase (XTH, previously known as xyloglucan endotransglycosylase, XET, EC 2.4.1.207), and endo-1,4-β-d-glucanase (EGase, EC 3.2.1.4) (Cutillas-Iturralde *et al.*, 1994; Luo, 2005). Genes encoding some of these enzymes are believed to be involved in fruit softening, such as *DkXTH1*/*2/8* (Nakatsuka *et al.*, 2011; Zhu *et al.*, 2013; Han *et al.*, 2016) and *DkExp3* (Zhang *et al.*, 2012), but little is known about the molecular aspects of persimmon fruit softening.

Unlike the regular softening that occurs during the ripening of persimmon fruit, the extreme softening that occurs post-deastringency is undesirable. As most of the commercialized persimmon belong to the astringent type (Yamada *et al.*, 1994; Wang *et al.*, 1997), which require the removal of astringency to reach an edible standard, this presents a major problem for the industry. Most persimmon post-deastringency fruit softening occurs rapidly after the removal of astringency. In recent years, addition of 1-methylcyclopropene (1-MCP), an inhibitor of ethylene perception, to high-CO_2_ treatment has been found to be effective for achieving deastringency while maintaining firmness for various persimmon cultivars (Harima *et al.*, 2003; Wang *et al.*, 2017). Initial attempts to investigate the transcriptional regulatory mechanisms of post-deastringency softening, with a limited range of ERFs, identified high-CO_2_/hypoxia responsive DkERF8/16/19 as activators of the promoter of *DkXTH9*, which encodes an enzyme related to hemicellulose degradation (Wang *et al.*, 2017). Transcripts of some AP2/ERF TFs have also shown significant correlations with fruit cell wall degradation and softening in various other fruits (Xie *et al.*, 2016), such as *MdCBF* in apple that activates *MdPG1* (Tacken *et al.*, 2010), kiwifruit *AdERF9* that acts as a suppressor of the *AdXET5* promoter (Yin *et al.*, 2010), and some other AP2/ERF genes in tomato (*SlAP2a*, Chung *et al.*, 2010; *Sl-ERF.B3*, Liu *et al.*, 2014). Apart from members of the AP2/ERF family, other TFs have also been found to play significant roles in tomato fruit softening, such as the MADS box TF *RIPENING INHIBITOR* (*RIN*) (Fujisawa *et al.*, 2013), *SlAN2* (an R2R3-MYB factor) (Meng *et al.*, 2015), and *SlNAC1* (NAM, ATAF1/ATAF2, CUC2) (Ma *et al.*, 2014). Hence, the larger-scale screening of TFs would very likely discover new regulators of post-deastringency softening in persimmon fruit.

In this study, RNA-seq was performed with persimmon fruit subjected to different treatments in order to search for more TFs beyond *ERF*s that are involved in this process. Comparative analyses between different treatments revealed multiple differentially expressed TFs. Dual luciferase assays and electrophoretic mobility shift assays indicated that DkNAC9 could physically bind and transactive the *DkEGase1* promoter, and this regulation could be enhanced by the presence of DkERF8/16, which has previously been identified as being involved in post-deastringent softening in persimmon (Wang *et al.*, 2017). However, our assays indicated that these were indirect regulators that functioned by interacting with DkNAC9, rather than with the target gene promoter. Our results thus provide valuable insights into the mechanisms of hypoxia-induced fruit softening.

## Materials and methods

### Plant material and treatments

Fruit of an astringent persimmon (*Diospyros kaki*) cultivar, ‘Jingmianshi’, were selected for this study and harvested from a commercial orchard in 2014 at Qingdao, Shandong, China. The experimental treatments have been described in detail in a previous study by Wang *et al.*, (2017). In brief, uniform mature fruit without disease or mechanical wounding were selected and divided into three batches that were treated in air-tight containers. (I) The first batch was treated with high CO_2_ for 1 d to remove astringency and to initiate rapid post-deastringency softening. (II) The second batch was treated with a combination of high CO_2_ and 1 μl·L^–1^ 1-MCP for 1 d, which achieved similar removal of astringency while maintaining the firmness of the fruit. (III) The third batch was sealed in air-tight containers for 1 d, as a control. After treatment, the fruit were transferred to storage in air at 20 °C. Collection of physiological data and details of sampling were as described by Wang *et al.* (2017), and the following experiments were all based on these samples.

### RNA extraction and cDNA synthesis

Total RNA extraction was conducted according to the methods described by Yin *et al.* (2012). Potential genomic DNA contamination was removed using a TURBO DNAse Kit (Ambion). A total of 1 μg RNA was used for cDNA synthesis from each sample, using an iScript^TM^ cDNA Synthesis Kit (Bio-Rad). All of the RNA extraction and cDNA synthesis reactions were performed with three biological replicates.

### Transcriptome analysis

Three batches of samples after 4 d in storage were selected to perform the RNA-seq, using the same RNA for RT-qPCR. The quality of the RNA for library construction was verified using a Qubit 2.0 Flurometer (Life Technologies) and a RNA Nano 6000 Assay Kit (Agilent Technologies). Library constructions, sequencing, and bioinformatics analyses were conducted by Novogene Bioinformatics Institute (Beijing). The clustering of the index-coded samples was performed on a cBot Cluster Generation System using TruSeq PE Cluster Kit v3-cBot-HS (Illumia) according to the manufacturer’s instructions. After cluster generation, the library preparations were sequenced on an Illumina Hiseq 4000 sequencing platform and paired-end reads were generated. For transcriptome analysis without a reference genome, transcriptome assembly was accomplished based on the left.fq and right.fq using Trinity (Grabherr *et al.*, 2011) with min_kmer_cov set to 2 by default and all other parameters set as default. Gene function was annotated based on the following databases: Nr (NCBI non-redundant protein sequences), Nt (NCBI nucleotide sequences), Pfam (Protein family), KOG/COG (eukaryotic Ortholog Groups/Clusters of Orthologous Groups of proteins), Swiss-Prot (a manually annotated and reviewed protein sequence database), KEGG (Kyoto Encyclopedia of Genes and Genomes), and GO (Gene Ontology). The iTAK software was used to predict TFs. Gene expression levels were estimated by using FPKM (fragments per kilobase of transcript sequence per millions base pairs sequenced; Trapnell *et al.*, 2010). Differential expression analysis of two conditions/groups was performed using the DESeq R package (1.10.1; Love *et al.*, 2014). The *P*-values were adjusted using the Benjamini–Hochberg approach for controlling the false discovery rate. An adjusted *P*-value<0.05 and |FoldChange|>5 were set as thresholds. KOBAS (Mao *et al.*, 2005) was used to test the statistical enrichment of differentially expressed genes in the KEGG pathways.

### RT-qPCR analysis

RT-qPCR was carried out on a CFX96 instrument (Bio-Rad), with SsoFast EvaGreen Supermix (Bio-Rad). The PCR reactions and procedures were performed as described by Wang *et al.* (2017). Gene-specific oligonucleotide primers were designed using Primer3 and are listed in Supplementary Table S1 at *JXB* online. The specificity of the primers was double-checked by melting-curve and PCR-product resequencing. The housekeeping gene *DkACT* (Min *et al.*, 2012) was chosen to monitor the abundance of mRNA. Real-time PCR reactions for each gene were performed at each sampling point of each treatment, with three biological replicates.

### Dual-luciferase assay

A dual-luciferase assay was conducted to test the *in vivo* regulatory effects of TFs on promoters of softening-related genes. Full-length genes were amplified with primers (Supplementary Table S2) and fused to the pGreen II 0029 62-SK vector (SK; Hellens *et al.*, 2005). Full-length of *DkERF* (*DkERF8/16/19*) genes had previously been constructed to SK by Min *et al.* (2012, 2014). Promoters of the cell wall metabolism-related genes (*Dkβ-gal1/4*, *DkEGase1*, *DkPE1/2*, *DkPG1*, and *DkXTH9/10*) were originally constructed by Wang *et al.* (2017), and were integrated into the pGreen II 0800-LUC vector (LUC; Hellens *et al.*, 2005).

All of the recombinant SK and LUC vectors were electroporated into *Agrobacterium tumefaciens* GV3101. The transfected *Agrobacterium* were grown in Luria–Bertani (LB) medium plates with 50 μg ml^–1^ kanamycin and 25 μg ml^–1^ gentamycin for 2 d and then re-streaked onto new LB plates for 1 d. *Agrobacterium* samples were resuspended in infiltration buffer (10 mM MES, 10 mM MgCl_2_, 150 μM acetosyringone, pH 5.6) and adjusted to OD_600_ of ~0.75. The cultures with the TF and promoter were then mixed (v/v, 10:1; for synergistic effect analysis, the TF1:TF2: promoter was 5:5:1; for gradient dilutions analysis, the TF1:TF2: promoter ratio changed from 10:0:1, 9:1:1 to 0:10:1) and infiltrated into leaves of tobacco (*Nicotiana benthamiana*) using syringes without needles. At 3d after infiltration, leaf discs were collected from the infiltrated areas and assayed using dual-luciferase assay reagents (Promega). Dual-luciferase assays were conducted with three replicates.

### Subcellular localization and phylogenetic analysis

35S-*DkERF8/16*-GFP (green fluorescent protein) and 35S-*DkNAC9*-GFP were transiently expressed in leaves of transgenic *N. benthamiana* with NLS-mCherry by *Agrobacterium*-mediated infiltration (GV3101) according to the method described by Xu *et al.* (2014). At 2 d after infiltration, the GFP signals of the leaves were imaged using a Zeiss LSM710NLO confocal laser-scanning microscope. The primers used for GFP constructions are listed in Supplementary Table S2.

The NAC gene (*DkNAC9*) was aligned with Arabidopsis *NAC* genes downloaded from the TAIR database (https://www.arabidopsis.org/). The alignment results were visualized using ClustalX (v.1.81) and FigTree (v.1.4.2).

### Electrophoretic mobility shift assay

The full-length *DkERF8*, *DkERF16*, and *DkNAC9* were inserted into the pGEX-4T-1 vector (GE), the constructs were then transformed into Rosetta (DE3) pLys bacteria (Novagen) by heat shock. Isopropyl β-d-1-thiogalactopyranoside (IPTG, 1 mM) was used to induce accumulation of the proteins at 16 °C for 20 h, then a GST-tag Protein Purification Kit (Beyotime Biotechnology) was used to purify the target proteins.

An electrophoretic mobility shift assay (EMSA) was performed using a LightShift Chemiluminescent EMSA kit (ThermoFisher Scientific). The probes used for this assay were synthesized and 3´-biotin labeled by HuaGene (Shanghai, China), and were mixed and annealed to the probe with its complementary chain to form a double strand.

### Bimolecular fluorescence complementation assay

The full-length of *DkERF8*, *DkERF16*, and *DkNAC9* were constructed into either C-terminal or N-terminal fragments of yellow fluorescent protein (YFP) vectors, using the primers listed in Supplementary Table S2 (the same primers were used in each case). All constructs were transiently expressed in leaves of transgenic *N. benthamiana* with NLS-mCherry by *Agrobacterium*-mediated infiltration (GV3101) according to previously published methods (Li *et al.*, 2016). The YFP fluorescence of the tobacco leaves was imaged 1 d after infiltration using a Zeiss LSM710NLO confocal laser-scanning microscope.

### Firefly luciferase complementation imaging assay

The full-length *DkERF8*, *DkERF16*, and *DkNAC9* were constructed into both the pCAMBIA1300-nLuc and pCAMBIA1300-cLuc (luciferase) vectors, using the primers listed in Supplementary Table S2. All constructs were transiently expressed in tobacco leaves by *Agrobacterium*-mediated infiltration (GV3101) as described previously (Li *et al.*, 2017*a*). The experimental combinations of TFs and the corresponding controls were injected into the same leaf and the luciferase activity of tobacco leaves was imaged 1 d after infiltration using a NightSHADE LB 985 imaging system (Berthold Technologies).

### Statistical analysis

Excel was used to conduct *t*-tests and ANOVA followed by least-significant difference (LSD) analyses. The figures were drawn using GraphPadPrism7 and Adobe Photoshop CS6.

### Accession numbers

The transcriptome data generated in this study have been submitted to the NCBI database with the BioProject ID PRJNA562104 and the GEO accession number GSE137588.

Sequence data from this paper are available at GenBank with the accession numbers KY849610, MH253881, MH621298, MK737978, MK737990, MK737999, MK738002, MK838487, MK838489, MK838490, and MK838492.

## Results

### Transcriptomic analysis and isolation of candidate TFs

**Fig. 1.**
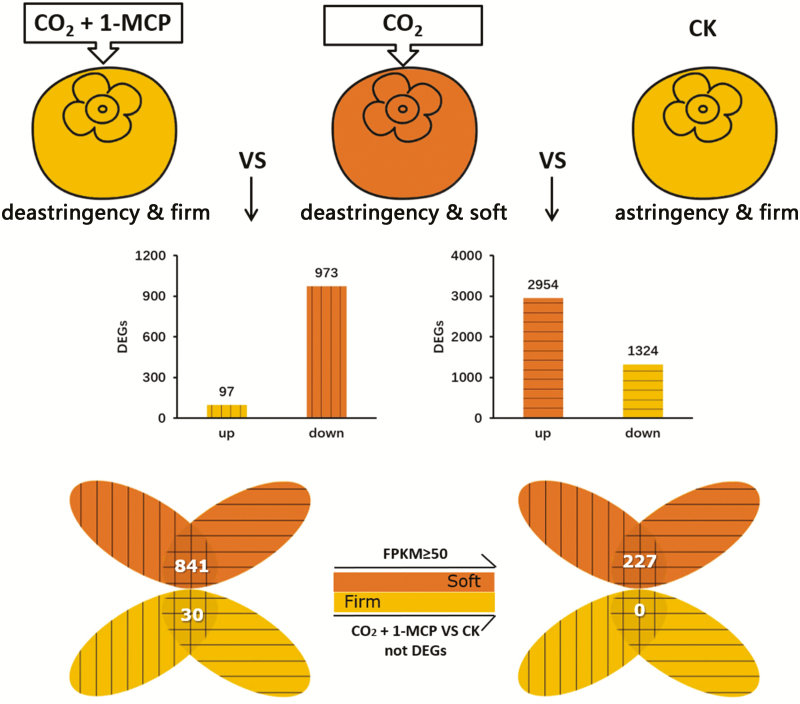
Identification by RNA-seq of differentially expressed genes (DEGs) responding to different treatments in post-harvest ‘Jingmianshi’ persimmon fruit. Mature fruit were treated with 95% CO_2_ (CO_2_), 95% CO_2_+1 μl l^–1^ 1-methylcyclopropene (CO_2_+1-MCP), or air (control, CK) for 1 d in air-tight containers, and then transferred to air. Samples were analysed 3 d after treatment. Orange indicates softening and yellow indicates that firmness was retained. The numbers of up- and down-regulated DEGs in each comparison are shown in the graphs. The Venn diagram to the left shows genes that had opposite trends in the two sets of comparisons. After removing the DEGs in the comparison between high-CO_2_+1-MCP and CK, 227 DEGs remained that had maximum FPKM values ≥50.

**Fig. 2.**
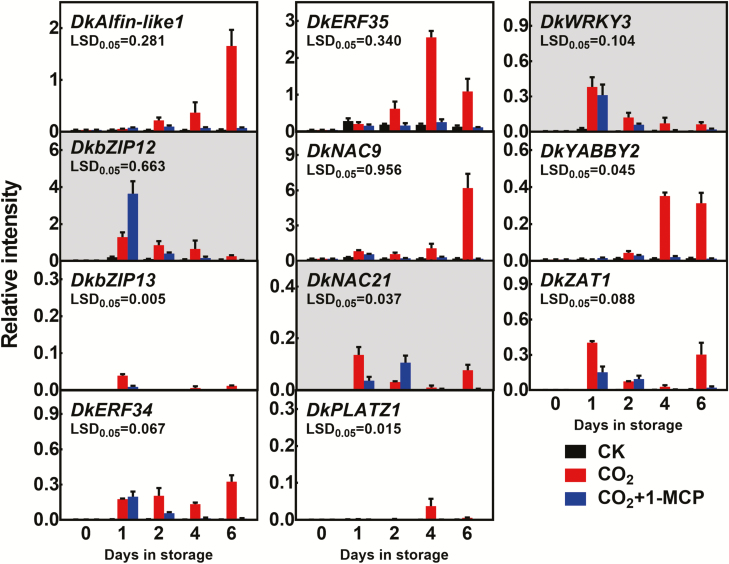
Expression of the differentially expressed transcription factors in ‘Jingmianshi’ persimmon fruit. Mature fruit were treated at 0 d in storage with 95% CO_2_ (CO_2_), 95% CO_2_+1 μl l^–1^ 1-methylcyclopropene (CO_2_+1-MCP), or air (control, CK) for 1 d in air-tight containers, and then transferred to air. Transcript levels were analysed by RT-qPCR. The three genes shown with grey backgrounds were discarded from further investigation, due to their lack of agreement with the RNA-seq results. Relative intensity is the expression relative to that of *DkACT*. Data are means (±SE) of three biological replicates.

High-CO_2_+1-MCP maintains firmness in persimmon fruit (Wang *et al.*, 2017), whilst high-CO_2_ causes rapid softening. Setting the adjusted *P*-value at <0.05 and |FoldChange| >5 as the thresholds, 2954 genes were found to be up-regulated by high-CO_2_ treatment (CO_2_) in ‘Jingmianshi’ persimmon fruit compared with the control (CK), and 1324 genes were down-regulated (Fig. 1). Comparison of the RNA-seq results for the high-CO_2_+1-MCP treatment (CO_2_+1-MCP) with the high-CO_2_ treatment alone indicated that 97 and 973 genes were up- and down-regulated, respectively. Firmness was one of parameters in the comparison (CO_2_ versus CK = soft versus firm; CO_2_+1-MCP versus CO_2_ = firm versus soft), and selection of genes that showed opposite trends in the two sets of samples enabled the number of potential candidate genes for post-deastringency softening to be reduced to 871 (Fig. 1). After removing the differentially expressed genes (DEGs) in the comparison between high-CO_2_+1-MCP and CK, the remaining DEGs with a maximum FPKM≥50 were then selected as a reference set (227 genes; Fig. 1, Supplementary Table S3) and their role in fruit softening was investigated. Of these 227 genes, 17 unigenes encoded putative TFs (Supplementary Table. S4). After full-length cloning and sequence analysis, 12 genes were obtained, namely *DkAlfin-like1* (*Alfin-like PHD finger*, MK738002), *DkbZIP12/13* (MK737990/MK838490), *DkERF34/35* (MK737978/MK838487), *DkNAC9/21* (MH253881/MK838489), *DkPLATZ1* (*plant AT-rich sequence- and zinc-binding*, MK838492), *DkWRKY3* (KY849610), *DkYABBY2* (MK737999), *DkZAT1* (*C2H2 zinc-finger*, MH621298) (Fig. 2), and the previously reported softening-related *DkERF8* (see Wang *et al.*, 2017).

Transcript levels of the 11 newly isolated TFs were verified by reverse-transcription quantitative PCR (RT-qPCR). The expression of *DkERF34/35*, *DkYABBY2*, *DkNAC9*, *DkAlfin-like1*, *DkPLATZ1*, *DkZAT1*, and *DkbZIP13* was substantially greater in high-CO_2_ than in the high-CO_2_+1-MCP and CK treatments (Fig. 2). *DkbZIP12* and *DkNAC21* were more abundant in the fruit treated with high-CO_2_+1-MCP at 1 d in storage (DIS) and 2 DIS respectively, and were not considered for further analysis. *DkWRKY3* showed no significant differences at 1 DIS and 2 DIS between high-CO_2_+1-MCP and high-CO_2_ and was also not considered further (Fig. 2).

### *In vivo* regulatory effects of selected TFs on promoters of genes involved in cell wall metabolism

**Fig. 3.**
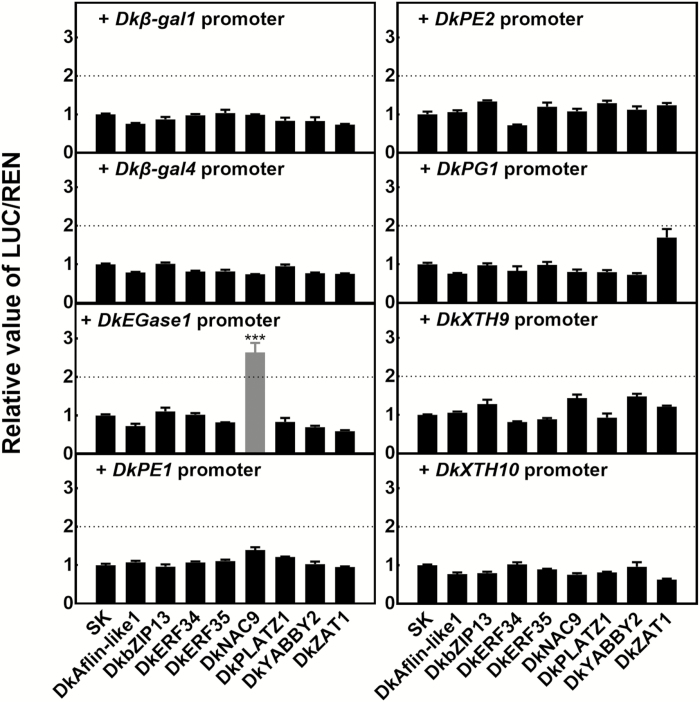
Regulatory effects of transcription factors on the promoters of genes related to cell wall metabolism in persimmon fruit. Dual-luciferase assays were used for analysis of regulatory effects. Values of the ratio of LUC/REN are expressed relative to that of the empty vector (SK) plus promoter, which was set as 1. Data are means (±SE) of three replicates and significant differences were determined using Student’s *t*-test: ***P<0.001.

To examine the potential regulatory effects of TFs on fruit-softening genes, the previously isolated promoters of eight genes were selected, namely *Dkβ-gal1/4*, *DkEGase1*, *DkPE1/2*, *DkPG1*, and *DkXTH9/10* (Wang *et al.*, 2017). Dual-luciferase assays indicated there was significant transactivation (2.6-fold) by DkNAC9 on the *DkEGase1* promoter, and very limited effects of the other TFs on the other gene promoters tested (Fig. 3).

**Fig. 4.**
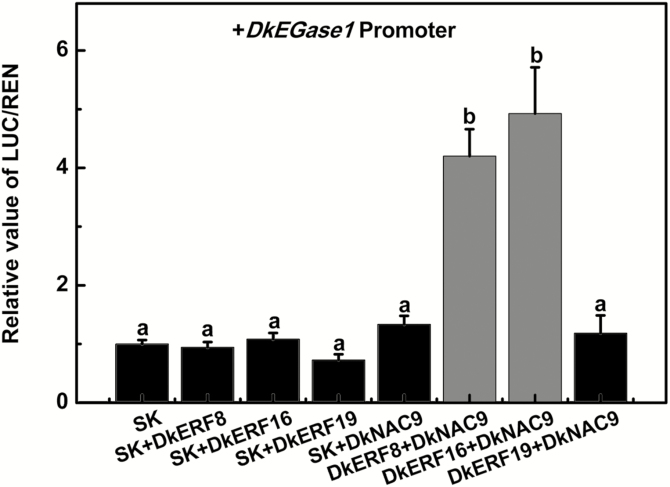
The synergistic effects of DkNAC9 and DkERFs on the promoter of *DkEGase1* in persimmon fruit. Dual-luciferase assays were used to test the synergistic effects of different combinations of transcription factors. Values of the ratio of LUC/REN are expressed relative to that of the empty vector (SK) plus promoter was, which was set as 1. Data are means (±SE) of three replicates. Different letters indicate significant differences between means as determined using ANOVA followed by LSD tests (*P*<0.05).

DkERFs 8, 16, and 19 have previously been shown to be positive regulators of the *DkXTH9* promoter, thus indicating a role in persimmon post-deastringency softening, but they have little effect on the *DkEGase1* promoter (Wang *et al.*, 2017). Using dual-luciferase assays, the synergistic effects of DkERFs and DkNAC9 on the *DkEGase1* promoter were investigated. As shown in Fig. 4, the combinations of DkERF8+DkNAC9 and DkERF16+DkNAC9 significantly enhanced the promoter activity of *DkEGase1*, by 4.2-fold and 4.9-fold, respectively, compared with the effects of individual DkERF8, DkERF16, or DkNAC9. However, the combination of DkERF19 and DkNAC9 failed to show any additive effect (Fig. 4).

The three positively acting TFs were fused to a GFP tag and expressed in tobacco leaves in order to examine their subcellular localization. All three genes produced strong signals in the nucleus, although some *DkERF8* signal was also found in plastids and *DkERF16* was also found in the cell membrane (Supplementary Fig. S1).

### Interactions between the TFs and the *DkEGase1* promoter

**Fig. 5.**
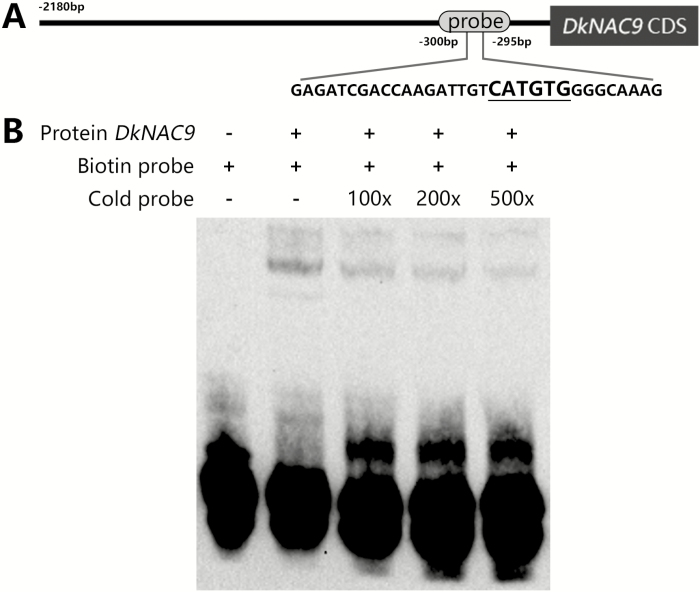
Analysis of the binding ability of DkNAC9 to the promoter of *DkEGase1*. (A) The probe sequence used for electrophoretic mobility shift assay (EMSA) in the *DkEGase1* promoter. The core sequence for NAC binding is underlined. (B) EMSA analysis. A 3´ biotin-labeled probe was used as the hot probe and the cold probe was without the 3´ biotin label.

In order to test the physical interactions between TFs and the *DkEGase1* promoter, EMSA was conducted. NAC family proteins have been reported to bind to the CATGTG motif (Tran *et al.*, 2004) and there was only one CATGTG motif in the *DkEGase1* promoter, which was located between approximately –300 bp and –295 bp (Fig. 5A). EMSAs indicated that DkNAC9 retarded the biotin-labeled probe and that the cold competitor probe (without biotin labeling) could reduce the band intensity (Fig. 5B). This indicated that DkNAC9 could physically bind to the CATGTG motif. *DkERF8* and *16* belong to the AP2/ERF family, and this family of proteins usually interact with a GCC box or DRE motif to regulate ethylene-responsive genes (Huang *et al.*, 2004; Zhang *et al.*, 2009). However, neither sequence was found in the *DkEGase1* promoter (from approximately –2180 to –1 bp). Some other rare motifs (e.g. ATCTA, CAACA) were also tried; however, the EMSA indicated that these ERF proteins could not interact with them (Supplementary Fig. S2).

### Protein–protein interactions between DkNAC9 and DkERF8 and DkERF16

**Fig. 6.**
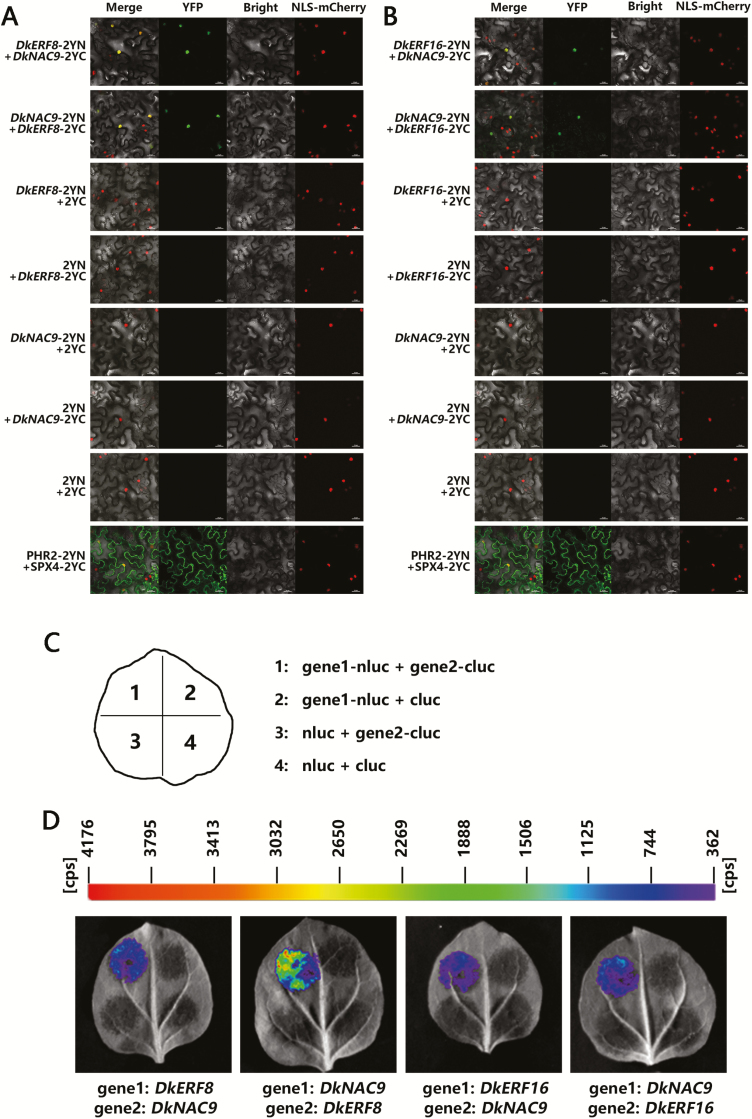
Protein–protein interactions between DkNAC9 and DkERF8/16 as analysed by bimolecular fluorescence complementation (BiFC) and luciferase complementation imaging (LCI) assays. Interactions between DkNAC9 and (A) DkERF8 and (B) DkERF16 as determined by BIFC. N- and C-terminal fragments of yellow fluorescent protein (YFP) (2YN and 2YC, respectively) were fused to the C-terminus of DkNAC9 and DkERF8/16, respectively. Combinations of 2YN or 2YC with the corresponding DkNAC9 and DkERF8/16 constructs were used as negative controls. PHR2-2YN + SPX4-2YC was used as the positive control. Fluorescence of YFP represents protein–protein interaction. Scale bars are 25 μm. (C) Schematic diagram of the injections for LCI, showing one experimental group and its three controls in a tobacco leaf. (D) Interactions between DkNAC9 and DkERF8/16 as determined by LCI. Combinations of nluc or cluc with the corresponding DkNAC9 and DkERF8/16 constructs were used as negative controls.

To clarify the synergistic effects of DkERF8 or DkERF16 with DkNAC9, we performed bimolecular fluorescence complementation (BiFC) assays using tobacco leaves. The results showed that co-expression of *DkERF8*-YFP^N^ and *DkNAC9*-YFP^C^ or *DkNAC9*-YFP^N^ and *DkERF8*-YFP^C^ both produced strong signals in the nucleus (Fig. 6A), while the negative combinations, such as *DkERF8*-YFP^N^ + YFP^C^, YFP^N^ + *DkNAC9*-YFP^C^, *DkNAC9*-YFP^N^**+** YFP^C^, YFP^N^ + *DkERF8*-YFP^C^, and YFP^N^**+** YFP^C^, all failed to exhibit any detectable fluorescence signal. Similar results were also found for interactions between DkERF16 and DkNAC9 (Fig. 6B) and the firefly luciferase complementation imaging (LCI) assay confirmed their interactions. All combinations of *DkERF8*-nluc + *DkNAC9*-cluc, *DkERF8*-cluc + *DkNAC9*-nluc, *DkERF16*-nluc + *DkNAC9*-cluc, or *DkERF16*-cluc + *DkNAC9*-nluc emitted fluorescence signals (Fig. 6C, D).

## Discussion

The molecular basis for softening has been widely investigated in various fruits and a few important regulators have been reported, such as MADS-RIN (Vrebalov *et al.*, 2002), *MaMYB3* (Fan *et al.*, 2018), and *AdDof3* (Zhang *et al.*, 2018). Most of these regulators mainly contribute to natural softening. It is well known that non-optimal environments, such as low temperature (Xu *et al.*, 2014) and water stress (Nakano *et al.*, 2002), also have a major influence on fruit texture; however, these have rarely been studied. Post-deastringency softening of persimmon is triggered by high-CO_2_ treatment (i.e. an extreme low-oxygen environment) and we used this to examine the environment–texture interaction.

### Transcriptomic analysis indicates multiple genes associated with post-deastringency softening

The underlying physiological, biochemical, and molecular effects of high-CO_2_ treatment on persimmon fruit deastringency have been extensively reported. Here, we found more than 4000 DEGs when comparing the high-CO_2_ treatment with controls (Fig. 1), which far exceeded the known number of deastringency regulators (~15 genes, namely *DkADH1/DkPDC2*, Min *et al.*, 2012; *DkERF9/10/19*, Min *et al.*, 2012, 2014; *DkERF18/21/22* and *DkMYB6/10*, Zhu *et al.*, 2018; *DkERF23/24/25* and *DkWRKY1/7*, Zhu *et al.*, 2019).

Wang *et al.* (2017) demonstrated that treatment with high CO_2_ combined with 1-MCP has similar effects on the maintenance of firmness in various cultivars of persimmon, which not only provides a potential practical application but also a means by which DEGs related to post-deastringency softening can be identified, by comparing the effects of high-CO_2_ alone versus high-CO_2_+1-MCP. Using RNA-seq, we obtained 12 full-length TFs among 227 DEGs, including the previously reported *DkERF8* (see Wang *et al.*, 2017). However, *DkERF16/19*, which have also been previously reported as being involved in fruit softening in persimmon, were not identified as being DEGs. We therefore individually examined the FPKM values of *DkERF16/19* and found that their transcript levels were up-regulated by high-CO_2_ and suppressed by high-CO_2_+1-MCP (Supplementary Fig. S3). We assume that the failure to identify them as DEGs was due to the variation among the three biological replicates in the initial screening.

In addition to the TFs, it was notable that the 227 DEGs were significantly enriched in anthocyanin biosynthesis, pentose and glucuronate interconversions, phenylpropanoid biosynthesis, monoterpenoid biosynthesis, starch and sucrose metabolism, and plant hormone signal transduction pathways (Supplementary Fig. S4). Our focus was to characterize softening-related genes, so we did not further investigate these other physiological changes. The carotenoid content for astringent persimmon fruit has previously been found to be higher after high-CO_2_ treatment (Plaza *et al.*, 2012), and the soluble sugar content has also been reported to change significantly during high-CO_2_ treatment, but without loss of sweetness (Ittah, 1993). However, the molecular mechanisms underlying these phenomena are still unclear, and in this respect our transcriptome data may also provide useful insights for future research. 

### Involvement of high CO_2_/hypoxia-responsive *DkNAC9* in regulating post-deastringency softening

**Fig 7.**
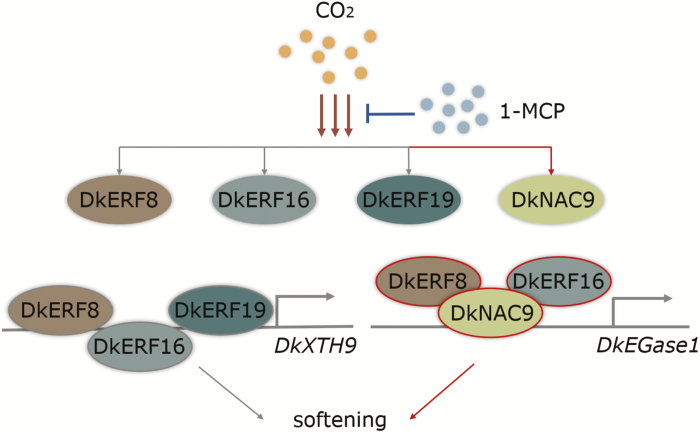
Proposed regulatory model for the role of transcription factors in persimmon fruit post-deastringency softening. Transcripts of *DkERF8/16/19* (Wang *et al.*, 2017) and *DkNAC9* are activated by high-CO_2_, but reduced when 1-methylcyclopropene (1-MCP) is also present. DkERF8/16/19 are involved in regulation of *DkXTH9*, which is related to cell wall metabolism (Wang *et al.*, 2017). DkNAC9 is a direct activator of the *DkEGase1* promoter, and DkERF8 and DkERF16 can interact with DkNAC9 to enhance this regulation. Red lines indicate new data from the current study and grey lines indicate previously published results.

Among the 11 newly isolated high-CO_2_/hypoxia-responsive TFs, only a NAC TF (DkNAC9) showed significant transactivation of the *DkEGase1* promoter, while the other TFs had little effect (Fig. 3). EMSA indicated a physical interaction of DkNAC9 with the CATGTG motif within the *DkEGase1* promoter (Fig. 5). These results point to the direct regulation of post-deastringency softening by DkNAC9, via regulation of *DkEGase1* (Fig. 7). The NACs belong to a family of plant-specific transcription factors that have been identified in various species (Aida *et al.*, 1997; Berger *et al.*, 2009; Zhong *et al.*, 2010), and most of the reported NAC TFs have been shown to have diverse functions in plant growth, development, and stress responses (He *et al.*, 2005; Christianson *et al.*, 2009; Xia *et al.*, 2010; Min *et al.*, 2015). Moreover, NACs such as *SlNAC1* (Ma *et al.*, 2014) and *Nor-like* genes (Gao *et al.*, 2018) have been reported to have roles in tomato ripening. However, the regulatory mechanisms of these NACs on fruit softening have not been specifically investigated. Our identification of DkNAC9 has not only revealed another regulator for post-deastringency softening of persimmon fruit, but also provides new clues with regards to regulation NAC-induced softening. Phylogenetic analysis indicated that *DkNAC9* is a homolog of *AtNAC102* (Supplementary Fig. S5), which is involved in the low-oxygen response in germinating seedlings of Arabidopsis (Christianson *et al.*, 2009). It remains unclear whether or not *AtNAC102* is related to cell wall metabolism. As *AtNAC102* is a hypoxic-response gene, we also examined the role of DkNAC9 in regulation of *DkADH1* and *DkPDC2*, two genes associated with anaerobic respiration (Min *et al.*, 2012), and the results indicated the *DkPDC2* promoter was transactivated by DkNAC9 (Supplementary Fig. S6). Thus, *DkNAC9* has a broader role in responses induced by low oxygen in persimmon fruit.

EGase is the enzyme that hydrolyses glucosidal bonds in the 1,4-*β*-glucan backbone, resulting in cellulose degradation. In tomato, the expressions of *EGase*s has been reported to increase during ripening (Gonzalez-Bosch *et al.*, 1996), and similar phenomena have been observed in other fruits, such as strawberry (Knee *et al.*, 1977) and avocado (O’Donoghue and Huber, 1992). Furthermore, the suppression of EGase results in firmer fruit and overexpression of EGase genes accelerates fruit softening (Brummell *et al.*, 1999). *DkEGase1* was the only coding gene for an EGase that reached the selection threshold in our previous study (Wang *et al.*, 2017), and therefore it may be regarded as the main contributor to the post-harvest decrease in cellulose. The role of DkNAC9 in *DkEGase1* regulation thus provides a new means by which the mechanism of fruit softening in persimmon may be elucidated.

### DkERF8 and DkERF16 are indirect regulators of the *DkEGase1* promoter via protein–protein interactions with DkNAC9

Our previous research indicated that three ethylene response-factor genes, *DkERF8*/*16*/*19*, are involved in regulation of the promoter of *DkXTH9* (Wang *et al.*, 2017). In our current study, we found synergistic interactions between DkNAC9 and both DkERF8 and DkERF16 (Fig. 4), which generated enhanced transactivation of the *DkEGase1* promoter. The differences in activation by DkNAC9 that can be seen in Figs 3 and 4 can be explained by the different concentrations used (Supplementary Fig. S7) with a DkNAC9:SK:promoter ratio of 5: 5: 1 (v:v:v) in Fig 4, compared with 10:0:1 in Fig 3. It can be assumed that DkERF8 and DkERF16 are indirect regulators, as all the EMSAs failed to generate band shifts (Supplementary Fig. S2). Moreover, BiFC and LCI assays all indicated potential protein–protein interactions between DkNAC9 and DkERF8 or DkERF16 (Fig. 6). Thus, it can be proposed that the *DkEGase1* promoter is directly regulated by DkNAC9, which is also the mediator for interactions with DkERF8/16 (Fig. 7). Our previous study indicated the direct regulation by DkERF8 and DkERF16 on the *DkXTH9* promoter (Wang *et al.*, 2017), and hence DkERF8/16 have dual direct and indirect functions in the regulation of two major cell wall genes (i.e. *DkXTH9* for hemicellulose degradation and *DkEGase1* for cellulose degradation) (Fig. 7).

A few ERFs have been reported as regulators for softening in kiwifruit (*AdERF9*; Yin *et al.*, 2010), apple (*MdCBF1*; Tacken *et al.*, 2010), tomato (*Sl-ERF.B3*; Liu *et al.*, 2014), and banana (*MaDEAR1*; Fan *et al.*, 2016). With regards to the regulation of ripening, protein complexes involving either ERFs or NACs have been reported in various fruit. For example, MaNAC1/2 interacts with the MaEIL5 transcription factor involved in ripening in banana (Shan *et al.*, 2012), a complex of CitNAC62 and CitWRKY1 contributes to citric acid degradation in citrus fruit (Li *et al.*, 2017*a*), and AP2/ERF genes have been reported as the partners for MYB transcription factors in the regulation of lignin (EjAP2-1, loquat; Zeng *et al.*, 2015) and anthocyanin (PyERF3, pear; Yao *et al.*, 2017). However, interactions between NAC and ERF transcription factors have rarely been reported for the regulation of fruit ripening. In conclusion, our present research not only suggests the involvement of DkNAC9 in regulating post-deastringency softening of persimmon fruit, but has also revealed the transcriptional complexes DkNAC9–DkERF8/16, which may also prove to be useful in understanding the roles of ripening-related NACs or ERFs in the regulation of fruit texture.

## Supplementary data

Supplementary data are available at *JXB* online.

Fig. S1. Subcellular localization of *DkERF8/16*-GFP and *DkNAC9*-GFP expressed in tobacco leaves.

Fig. S2. Analysis of the binding ability of DkERF8/16 to the promoter of *DkEGase1*.

Fig. S3. The FPKM values of previously reported *DkERF*s in response to CO_2_ and CO_2_+1-MCP treatments in the ‘Jingmianshi’ cultivar.

Fig. S4. KEGG enrichment analyses of DEGs in response to the different treatments.

Fig. S5. Phylogenetic analyses of persimmon NAC genes.

Fig. S6. Regulatory effects of DkNAC9 with or without DkERF8/16 on the promoters of *DkADH1* and *DkPDC2*.

Fig. S7. Effects of different dilutions of DkNAC9 on the *DkEGase1* promoter.

Table S1. Sequences of the primers used for real-time PCR.

Table S2. Sequences of the primers used for vector construction.

Table S3. The expression of 227 DEGs in response to the different treatments.

Table S4. Annotation of the 227 DEGs in response to the different treatments.

eraa009_suppl_supplementary_figures_S1_S7_tables_S1_S2

eraa009_suppl_supplementary_table_S3

eraa009_suppl_supplementary_table_S4

## Abbreviations

1-MCP: 1-methylcyclopropene
BiFC: bimolecular fluorescence complementation
DEGs: differentially expressed genes
DIS: days in storage
EGase: endo-1,4-*β*-d-glucanase
EMSA: electrophoretic mobility shift assay
ERF: ethylene response factor
LCI: luciferase complementation imaging
TFs: transcription factors
XTH: xyloglucan endotransglycosylase/hydrolase
YFP: yellow fluorescent protein
