# Supplementary material for: High CO2/hypoxia-induced softening of persimmon fruit is modulated by DkERF8/16 and DkNAC9 complexes
Source: J Exp Bot. 2020 Jan 12;71(9):2690–700. doi: 10.1093/jxb/eraa009 (PMC7210769; doi:10.1093/jxb/eraa009)
Supplement: eraa009_suppl_supplementary_figures_S1_S7_tables_S1_S2 [file eraa009_suppl_supplementary_figures_s1_s7_tables_s1_s2.pdf]

Fig. S1

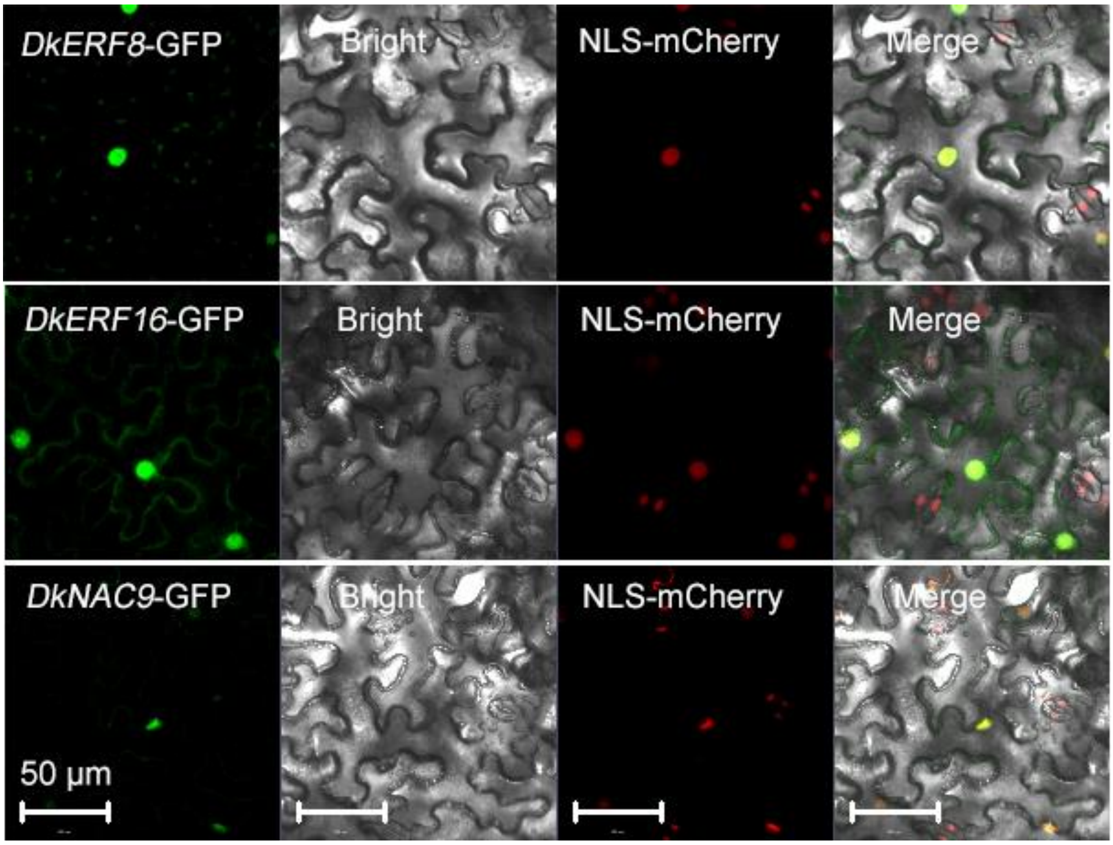

Supplementary Figure S1. Subcellular localization of *DkERF8/16*-GFP and *DkNAC9*-GFP in tobacco leaves.

Fig. S2

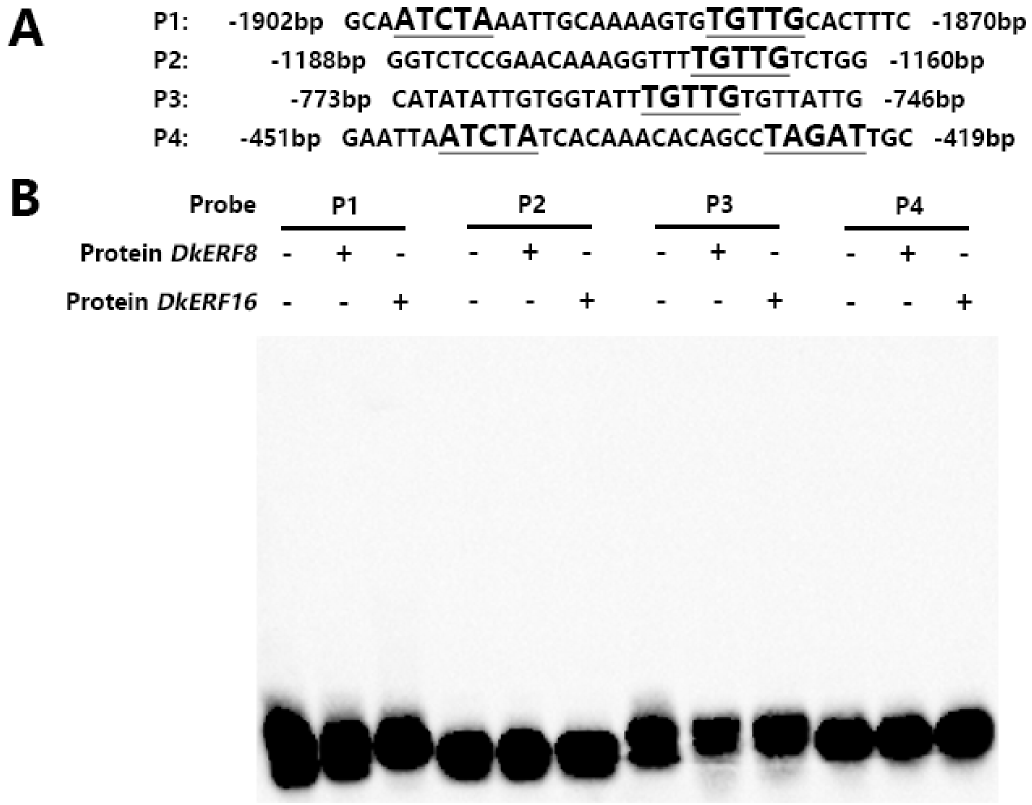

Supplementary Figure S2. Analysis of the binding ability of DkERF8/16 to the promoter of *DkEGase1*.

Fig. S3

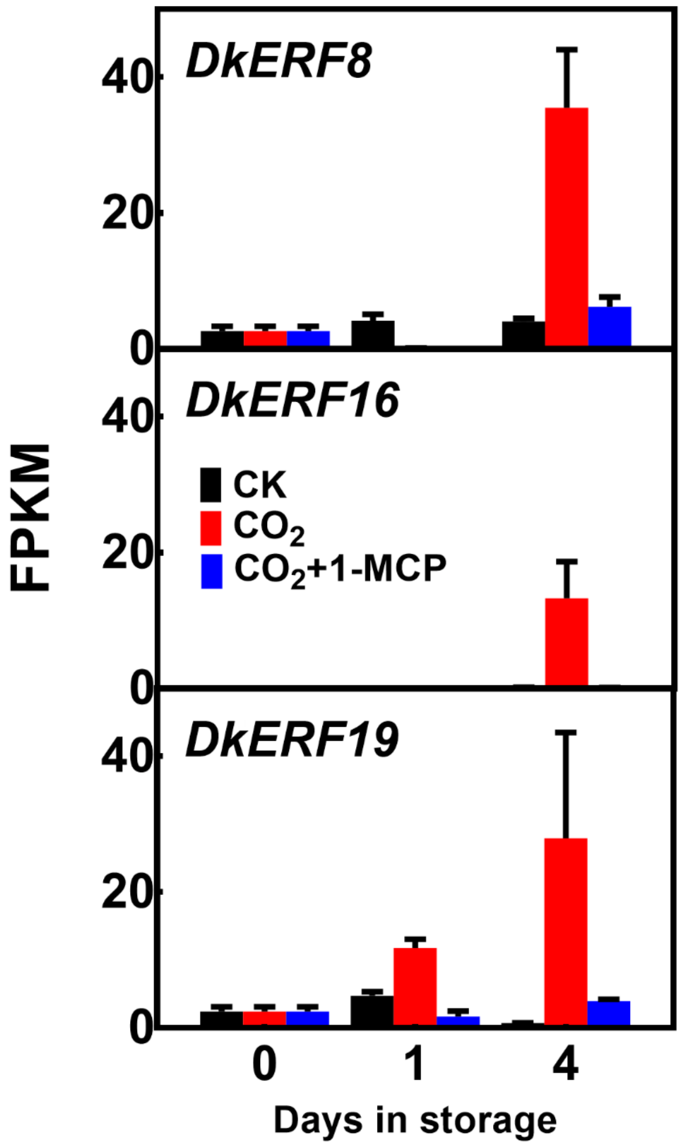

Supplementary Figure S3. The FPKM of previous reported *DkERFs* in response to CO_2 and CO_2 + 1-MCP treatments in 'Jingmianshi' cultivar.

Fig. S4

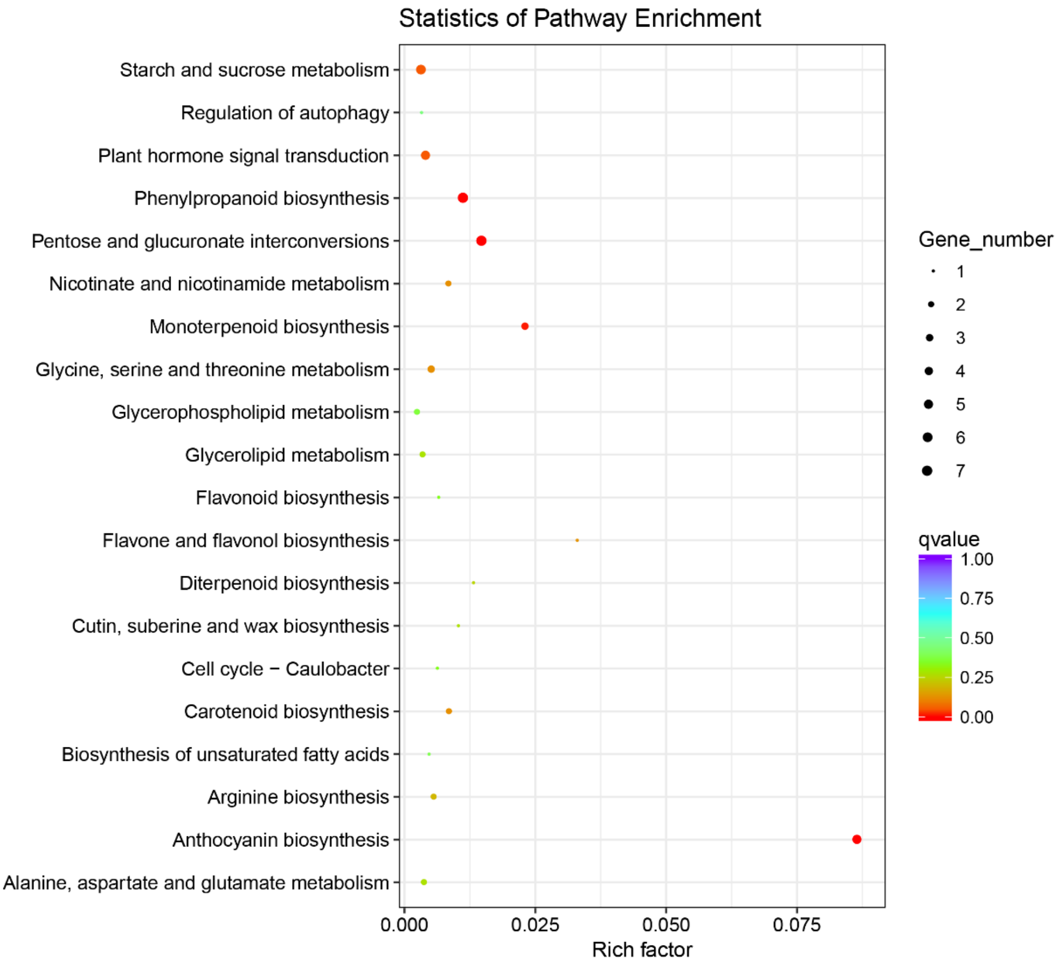

Supplementary Figure S4. KEGG enrichment analyses of DEGs in response to different treatments.

Fig. S5

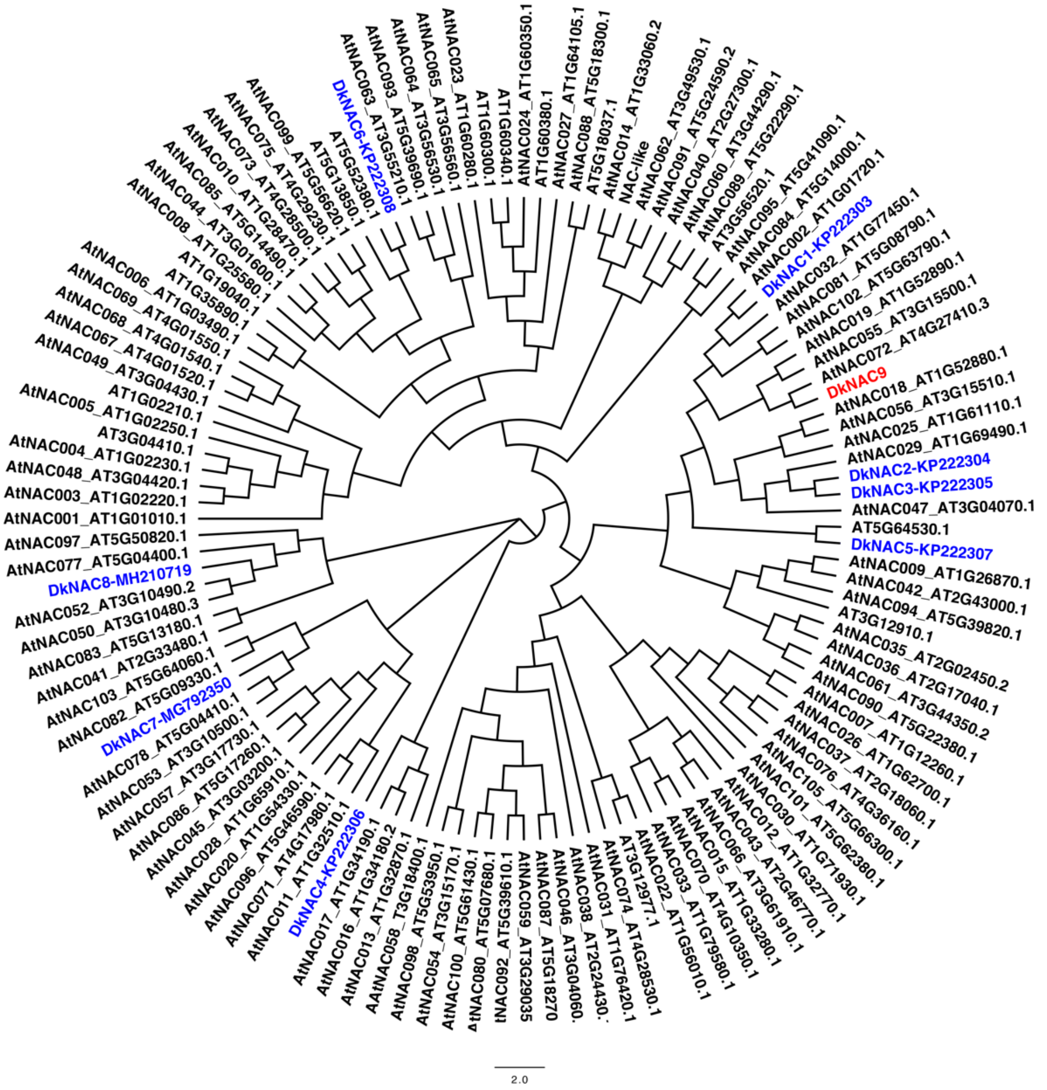

Supplementary Figure S5. Phylogenetic analyses of persimmon NAC genes.

Fig. S6

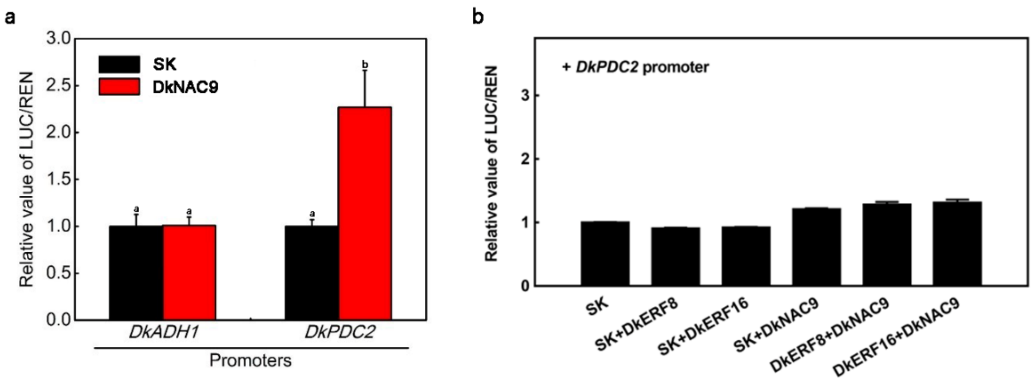

Supplementary Figure S6. Regulatory effects of DkNAC9 with or without DkERF8/16 on the promoters of *DkADH1* and *DkPDC2*.

Fig. S7

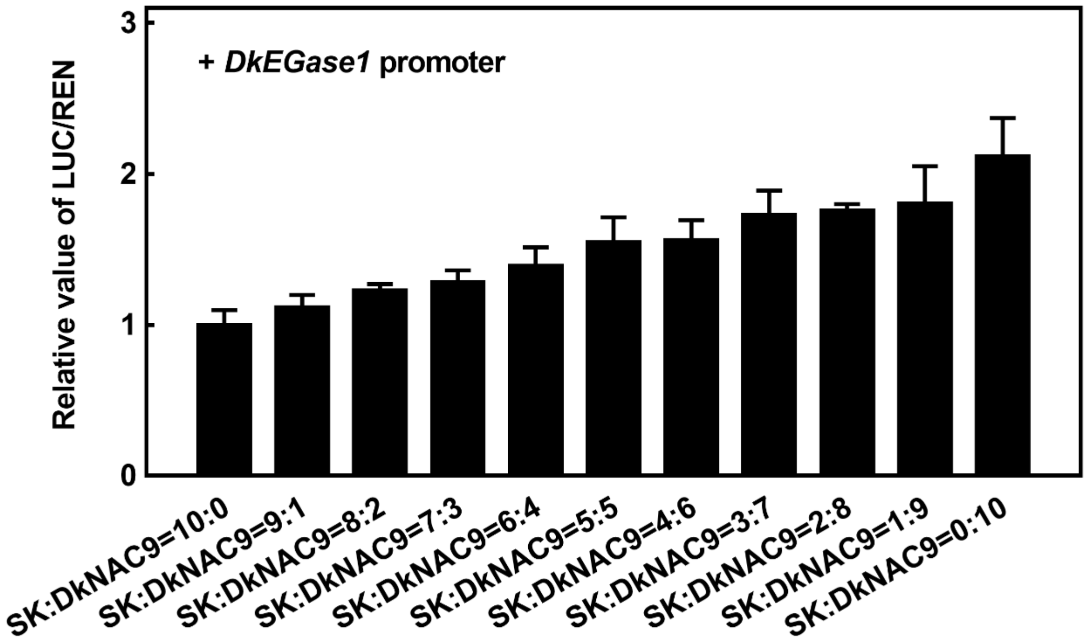

Supplementary Figure S7. Effects of different dilutions of DkNAC9 on *DkEGase1* promoter.

Supplementary Table S1. Sequences of the primers used for real time PCR.

Gene	Methods used	Primers (5´-3´)
DkERF35	Q-PCR (FP)	GTGGGTACCAACACAATGAATC
DkERF35	Q-PCR (RP)	AGCATCCAGAGATCAACCAAGT
DkYABBY2	Q-PCR (FP)	CAAAGCTGGAAATCCCGATA
DkYABBY2	Q-PCR (RP)	GATCAGGCAAAAGTCCGAAA
DkbZIP12	Q-PCR (FP)	CATGGCAGTCGAAACAGAGA
DkbZIP12	Q-PCR (RP)	GGCAGAGACTGGTGAGGAAG
DkNAC9	Q-PCR (FP)	GGGCTGTAGAGGAGGAGGTT
DkNAC9	Q-PCR (RP)	TTTTCCTTCACTGCCGAAAC
DkAlfin-like1	Q-PCR (FP)	GACTGCCAAGAAACAAGCAA
DkAlfin-like1	Q-PCR (RP)	ACAAGCCCCACACAAGGTAT
DkWRKY3	Q-PCR (FP)	CGGATGTTCAAGAGTTTTTGG
DkWRKY3	Q-PCR (RP)	GATGGCGATCATCTTGGATAA
DkbZIP13	Q-PCR (FP)	GCTGTAGCGATGGAGGAGTC
DkbZIP13	Q-PCR (RP)	GACGGGTTGGTTCAGGTAGA
DkPLATZ1	Q-PCR (FP)	GGGTTGTAAGCTTGCAGGAA
DkPLATZ1	Q-PCR (RP)	ATGTCTTGTTGCGACCCTTC
DkNAC21	Q-PCR (FP)	GGAAGAATGCCATCCCAGTA
DkNAC21	Q-PCR (RP)	AATTCTTCCACCCTGCATTG
DkERF34	Q-PCR (FP)	CGGTTCGTGAAGGAGAAGAG
DkERF34	Q-PCR (RP)	CTGACGGCACCTTGAATATG
DkZAT1	Q-PCR (FP)	TGTAACCGGGAGTTTTCGTC
DkZAT1	Q-PCR (RP)	TTCTCGTAGGGCGTCAAGTT

Supplementary Table S2. Sequences of the primers used for vector construction.

Gene	Methods used	Primers (5´-3´)
DkERF35	SK vector construction (FP)	GCTCTAGAACTAGTGGATCCATGTGCGG TGGGGCGATCAT
DkERF35	SK vector construction (RP)	ATAAGCTTGATATCGAATTCTTAGAACC CCAGGTGGTGATG
DkYABBY2	SK vector construction (FP)	GCTCTAGAACTAGTGGATCCATGTCCTC CTCATCATCCG
DkYABBY2	SK vector construction (RP)	ATAAGCTTGATATCGAATTCTTAGTAGG GGGATAGCC
DkNAC9	SK vector construction (FP)	ATAGCGGCCGCATGGGATTGCCGGTGTC AGACCC
DkNAC9	SK vector construction (RP)	CTGACTAGTTTATTTCTCTGTTTTCCTTC ACTGC
DkAlfin-like1	SK vector construction (FP)	GCTCTAGAACTAGTGGATCCATGGATGG CGGAGGACCGTAC
DkAlfin-like1	SK vector construction (RP)	ATAAGCTTGATATCGAATTCTCAAGGCC GCGCTCTCTTG
DkbZIP13	SK vector construction (FP)	GCTCTAGAACTAGTGGATCCATGGCCTC TTCAAGCC
DkbZIP13	SK vector construction (RP)	ATAAGCTTGATATCGAATTCTCAGGACA TGATCATG
DkPLATZ1	SK vector construction (FP)	GCTCTAGAACTAGTGGATCCATGCGGGT TCCGCCATG
DkPLATZ1	SK vector construction (RP)	ATAAGCTTGATATCGAATTCCTAGGCGC CAAGCGG
DkERF34	SK vector construction (FP)	GCTCTAGAACTAGTGGATCCATGGAAG GCAGTTGCACAG
DkERF34	SK vector construction (RP)	ATAAGCTTGATATCGAATTCTTACAAAG CCACTCCAATG
DkZAT1	SK vector construction (FP)	GCTCTAGAACTAGTGGATCCATGACGGT GTTGACGAAG
DkZAT1	SK vector construction (RP)	ATAAGCTTGATATCGAATTCTCATCGGG CAATTTGCAAC
DkNAC9	GFP vector construction (FP)	ACGGGGGACGAGCTCGGTACCATGGGA TTGCCGGTGTCAGACC
DkNAC9	GFP vector construction (RP)	GCCCTTGCTCACCATGTCGACCTGCCGA AACCCGAATTGTC
DkERF8	GFP vector construction (FP)	ACGGGGGACGAGCTCGGTACCATGCAA GCAAGCAACGATATTCG
DkERF8	GFP vector construction (RP)	GCCCTTGCTCACCATGTCGACATTAGCA AGAACTTCCCAA

Gene	Methods used	Primers (5´-3´)
DkERF16	GFP vector construction (FP)	ACGGGGGACGAGCTCGGTACCATGGCT AGACCTCAGCAGCGA
DkERF16	GFP vector construction (RP)	GCCCTTGCTCACCATGTCGACCATAGCC TGATGAGATGGAAC
DkNAC9	GST vector construction (FP)	GGATCTGGTTCCGCGTGGATCCATGGGA TTGCCGGTGTCAG
DkNAC9	GST vector construction (RP)	CGATGCGGCCGCTCGAGTCGACTCACTG CCGAAACCCGAATTG
DkERF8	GST vector construction (FP)	GGATCTGGTTCCGCGTGGATCCATGCAA GCAAGCAACGATATTC
DkERF8	GST vector construction (RP)	CGATGCGGCCGCTCGAGTCGACCTAATT AGCAAGAACTTCCCAAATC
DkERF16	GST vector construction (FP)	GGATCTGGTTCCGCGTGGATCCATGGCT AGACCTCAGCAGCG
DkERF16	GST vector construction (RP)	CGATGCGGCCGCTCGAGTCGACTCACAT AGCCTGATGAGATGG
DkNAC9	YFP vector construction (FP)	TACGAACGATAGTTAATTAATATGGGAT TGCCGGTGTCAGA
DkNAC9	YFP vector construction (RP)	TCCTCCACTAGTGGCGCGCCCCTGCCGA AACCCGAATT
DkERF8	YFP vector construction (FP)	TACGAACGATAGTTAATTAATATGCAAG CAAGCAACGATAT
DkERF8	YFP vector construction (RP)	TCCTCCACTAGTGGCGCGCCCATTAGCA AGAACTTCCCAAATC
DkERF16	YFP vector construction (FP)	TACGAACGATAGTTAATTAATATGGCTA GACCTCAGCAGCG
DkERF16	YFP vector construction (RP)	TCCTCCACTAGTGGCGCGCCCCATAGCC TGATGAGATGGAACA
DkNAC9	nLuc vector construction (FP)	ACGGGGGACGAGCTCGGTACCATGGGA TTGCCGGTGTCAG
DkNAC9	nLuc vector construction (RP)	CGCGTACGAGATCTGGTCGACCTGCCGA AACCCGAATTG
DkERF8	nLuc vector construction (FP)	ACGGGGGACGAGCTCGGTACCATGCAA GCAAGCAACGATATTC
DkERF8	nLuc vector construction (RP)	CGCGTACGAGATCTGGTCGACATTAGCA AGAACTTCCCAAATC
DkERF16	nLuc vector construction (FP)	ACGGGGGACGAGCTCGGTACCATGGCT AGACCTCAGCAGCG
DkERF16	nLuc vector construction (RP)	CGCGTACGAGATCTGGTCGACCATAGCC TGATGAGATGG
DkNAC9	cLuc vector construction (FP)	TACGCGTCCCGGGGCGGTACCATGGGAT TGCCGGTGTCAG

Gene	Methods used	Primers (5´-3´)
DkNAC9	cLuc vector construction (RP)	ACGAAAGCTCTGCAGGTCGACTCACTGCCGAAACCCGAATTG
DkERF8	cLuc vector construction (FP)	TACGCGTCCCGGGGCGGTACCATGCAAGCAAGCAACGATATTC
DkERF8	cLuc vector construction (RP)	ACGAAAGCTCTGCAGGTCGACCTAATTAGCAAGAACTTCCCAAATC
DkERF16	cLuc vector construction (FP)	TACGCGTCCCGGGGCGGTACCATGGCTAGACCTCAGCAGCG
DkERF16	cLuc vector construction (RP)	ACGAAAGCTCTGCAGGTCGACTCACATAGCCTGATGAGATGG
